# Structure–Property Functions of Inorganic Chemical Binders for Refractories

**DOI:** 10.3390/ma14164636

**Published:** 2021-08-17

**Authors:** Vanessa Hopp, Ali Masoudi Alavi, Dominik Hahn, Peter Quirmbach

**Affiliations:** Faculty for Natural Science, Campus Koblenz, University of Koblenz-Landau, 56070 Koblenz, Germany; masoudi@uni-koblenz.de (A.M.A.); dominikhahn@uni-koblenz.de (D.H.); pquirmbach@uni-koblenz.de (P.Q.)

**Keywords:** inorganic chemical binders, refractories, phosphates, water glasses

## Abstract

For refractory application, amongst others, inorganic chemical binders are used to shape and process loose, unpacked materials. The binder influences the chemical composition within the ceramic body during setting, aging and firing and thus the finally reached properties of the refractory material. For an effective design of tailored materials with required properties, the mode of action of the binder systems should carefully be investigated. A combination of both structure analysis techniques and macroscopic property investigations proved to be a powerful tool for a detailed description of structure–property correlations. This is shown on the basis of X-ray powder diffraction and nuclear magnetic resonance spectroscopy analyses combined with observation of (thermo)mechanical and chemical investigations.

## 1. Introduction

Ceramics obtain their typical properties such as strength and hardness specifically through sintering of ceramic particles by heat treatment above their sintering temperature [1]. In practice, therefore, there is usually a need to increase the green strength of the ceramic body to enable components to be shaped and processed. For this purpose, binder additives are used which lead to the bonding of loose, unpacked materials such as monolithic refractory ceramics [2].

In principle, binders act through adhesive and cohesive forces. Cohesive forces take effect especially within the binder system itself, while adhesion results from bonding forces at the interface between the binder and the connected material, e.g., ceramic material [3]. However, depending on the type of binder used, their modes of action and effective operating temperatures differ significantly. For example, temporary (organic) binders or permanent (hydraulic or chemical) binders are applied both within the refractory industry production and other ceramic materials sectors.

Besides the major technically advances of binders, considerations must be made in respect to binders being able to decisively influence the chemical composition within the ceramic body composition during setting, aging and firing. Binders may actively enter into interactions and chemical reactions with ceramic components which, consequently, has a great influence on materials properties, e.g., strength, thermal properties or resistance to chemical attack (erosion, wear, corrosion). In particular, it must be considered that especially the bonding phase, as the interphase between the grains of the refractory material, shows high sensitivity to physical-chemical and mechanical impact. For refractory manufacturers, selecting a suitable binder additive is therefore of decisive importance to increase production efficiency and to set the desired product quality.

Therefore, the evaluation of influences of the binder additive (1) on the property development of the ceramic (e.g., mechanical, chemical and thermal stability) is essential and, furthermore, (2) mineralogical and chemical structural changes within the ceramic composition caused by the binder interactions must be observed analytically. Simultaneous monitoring and correlating of changes in materials properties and structures leads to a fundamental understanding of bonding mechanism and a targeted use of binders.

## 2. The Principle of Binders

Bonding systems for refractory application can be distinguished into cold bonding systems and hot bonding types. Hot bonding describes mainly the development of a bonding phase due to sintering processes of the ceramic particles at high temperatures. This so-called ceramic bonding can replace other types of binders in the case of temperature treatment of the material. Cold bonding systems always require the addition of a binder and are classified as organic, hydraulic and chemical binders (see overview in Table 1) [4].

The group of organic binders (e.g., resins, polymers) are mainly applied as temporary binder systems. After the firing process at high temperatures, no organic residues remain in the refractory material due to the decomposition and burning of the organic species.

The bonding effect of hydraulic binders is based on the formation of interlocking hydrate phases. In the refractory sector, mainly calcium aluminate cement phases are applied [4,5]. In special cases the application of ρ-alumina as a source for hydratable alumina are present as cold bonding agents [6].

Another class of binders for industrial applications are the inorganic chemical binders. This group includes all the types, whose bonding effect is based on chemical reactions either with ceramic particles or within the binder itself [4,7]. Examples of chemical binders are colloidal sols like silica, alumina and sols like water glass and phosphate binders. Phosphate binders and water glasses can be applied both as liquid and solid-state basic materials. In the case of phosphates as chemical binders, mainly phosphoric acid (H_3_PO_4_) or metal phosphate salts, e.g., mono aluminum phosphate (MALP) are applied [8]. In the group of colloidal sols, mainly water glasses, for refractories especially sodium water glasses, are present.

As a requirement for the bonding ability of inorganic chemical binders in refractory applications only substances, that are able to generate a three-dimensional network structure either by chemical activation or due to a drying process are suitable. Therefore, glass-forming structures like silicates and phosphates are preferred as bonding agents.

Silicon is situated in the fourth group of the periodic table of the elements which causes a high variety of bonding possibilities. The oxides of silicon, so-called silicates, are specified as network forming structures by linkage of SiO_4_ tetrahedrons as fundamental units. The oxygen can perform as bridging oxygen and is therefore essential for the development of a three-dimensional network. In similar way, the oxides of phosphorus are network formers, as well. Phosphorus is situated in the fifth group of the periodic table of the elements and thus shows another connectivity compared to silicon; however, due to the radius of phosphorus cations, which is very similar to silicon, the P^5+^ cation forms tetrahedral units. In this case a double bond to one of the oxygen atoms is formed, causing a disconnecting point.

## 3. Setting and Bonding Mechanism

In the group of inorganic chemical binders, the use of phosphate and water glass binders brings advantages for industrial refractory applications. Phosphate binders generate great green strength of shaped and unshaped refractories leading to user-friendly handling for example while demolding and transportation. Water glass binders are based on a sol-gel transformation that allows running the setting process at room temperature.

### 3.1. Phosphates

**Figure 1:**
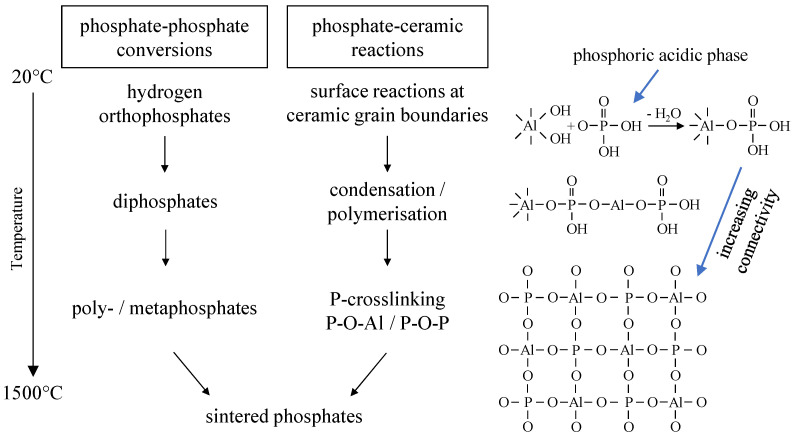
Overview of the bonding mechanism of inorganic phosphate binders in an alumina-based ceramic application as a function of temperature.

The phosphate bonding mechanism generally consists of three reaction steps summarized in Figure 1 [9,10,11]. Bonding in a first step is initiated by an acid–base reaction between the phosphates and the ceramic oxides during setting in the presence of process-related water. This includes dissolution processes of both the ceramic oxides (formation of aquasols, M-OH) and the phosphates (phosphoric acidic phase). Therefore, initiation strongly depends on the water-solubility of the phosphates as well as pH-values. The acid–base reactions result in the formation of network-forming phosphates (e.g., aluminum phosphates, crystalline and amorphous), that lead to adhesive and cohesive bonding forces. In a second reaction step the P-crosslinking of the bonding phase and thus the bonding effect is increased by temperature treatment below T < 800 °C. This network formation is mainly due to condensation and polymerization reactions of the phosphates. In a third reaction step (T > 800 °C) high-temperature reactions result in the formation of sintered phosphates with higher crystallinity, mostly ortho phosphates. In addition, further reactions between the phosphate species and the ceramic oxides occur with frequently cation exchange reactions. Simultaneously, sintering of ceramic oxides takes places causing the phosphate bonding to merge into ceramic bond, thus achieving permanent bonding.

Additionally, it should be pointed out that only certain phosphate structures (network-forming, e.g., aluminum phosphates) can generate bonding effects. The formation of bonding networks (as a consequence of reactions with ceramic oxides) is crucial for achieving cohesion and adhesion in the bonding phase/matrix. Accordingly, bonding capacities cannot be attributed to inert phosphates that do not react or interact with the ceramic oxides at low temperatures (e.g., calcium or magnesium phosphates). The conversion schemes of these phosphates (hydrogen orthophosphates—diphosphates—poly- or metaphosphates) do not generate bonding networks.

### 3.2. Silicates

**Figure 2:**
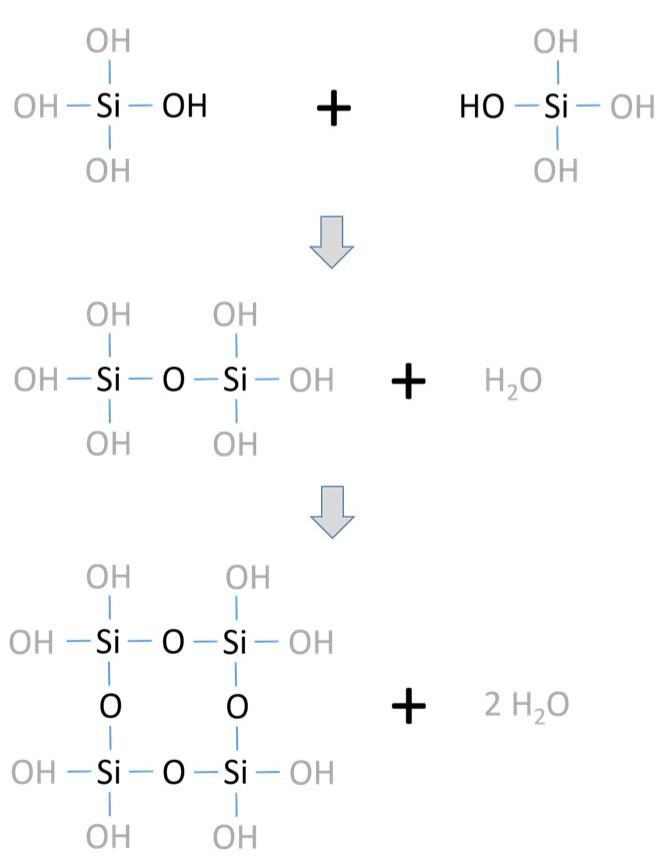
Overview of the network formation in silicate systems due to the polycondensation of silanol groups to siloxanes.

As silicate systems of inorganic binders mainly water glasses like sodium water glasses are applied either as a colloidal solution or as a dry powder. The setting of the water glass binders can be initiated by different reaction routes depending on the desired properties. The simplest setting is activated by heat. Due to the thermal dehydration of the water glass systems polycondensation occurs forming an amorphous three-dimensional binder framework (see overview in Figure 2).

**Figure 3:**
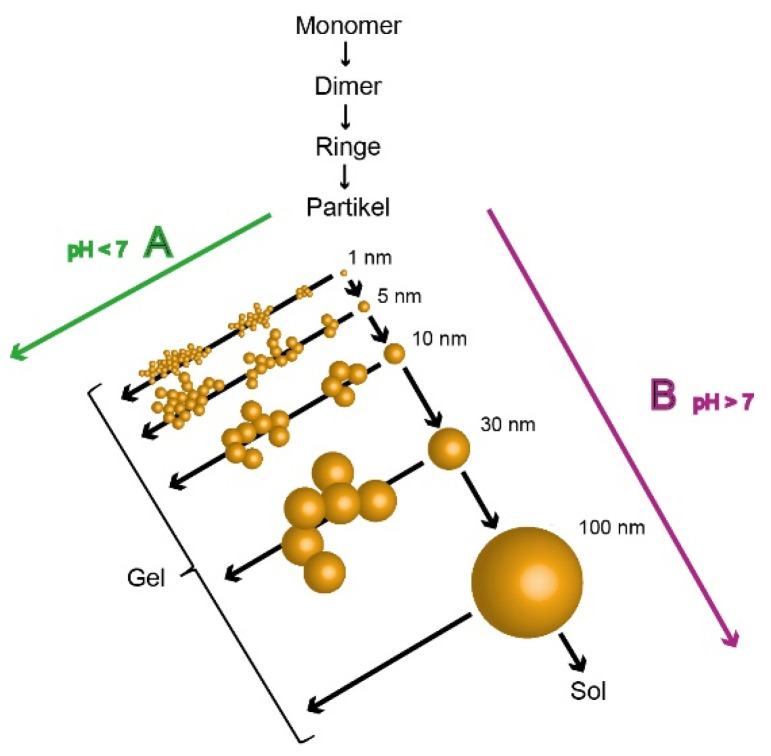
Influence of pH value on the particle size and network formation of silicates. Adapted from [14].

To reach an increased green strength and an improved thermal stability of the refractory system, the setting mechanism is activated chemically, for example due the use of inorganic phosphates, mainly aluminum phosphates as hardening agents. In this case, the setting mechanism occurs in a multi-step reaction including (a) an ion-exchange reaction between the alkali ions of the water glass and the metal ions of the phosphate hardener, (b) the formation of crystalline alkali phosphate structures and (c) an acid–base reaction. The drop of pH value lead to a reduction of the electrochemical stabilization of the sol, thus causing the silanol groups to condensate to siloxanes. In Figure 3 the influence on the particle size of the silicate structures and the network formation is illustrated in terms of the pH value. With an increase in pH value the particle size of colloidal silicates is increased forming a sol state, which is mainly stabilized due to electrostatic forces by the negative surface charges based on the DVLO theory [12,13]. With lowering pH value, the stabilization decreases and the polycondensation of the cross-linking of the silicate particles occurs forming amorphous three-dimensional frameworks to a gel state. In the case of water glasses the alkali modulus defines the pH value due to the alkaline nature of the M_2_O content [14].

In this multi-reaction setting all of the single steps are in competition to each other and have a massive impact on the properties (e.g., change in mechanical strength determined by cold bending strength, Young’s modulus) of the refractory system. Therefore, a good insight into the setting mechanism is the key step to the adjustment of tailored sample properties based on the binder performance.

## 4. Measurements of Structure and Property Information

Due to the enormous impact of the bonding matrix structure on the properties of the refractory material, the chemical and mineralogical structure investigations of the binders itself as well as the binder–ceramic compound have a high relevance in the process of material design. For determining the chemical and/or mineralogical structure of materials a wide range of different analytical methods are quite well known in the field of material science. In the case of the three-dimensional phosphate or silicon-based binder matrices, the appropriate combination of different measuring methods is essential for an overarching description. This is mainly due to the existence of both amorphous and crystalline phases. A strong base for the structure determination of phosphate or silicon-based binder matrices is therefore the combination of the complementary X-ray powder diffraction and nuclear magnetic resonance spectroscopy.

X-ray powder diffraction (PXRD) is a standard and widely used method for the investigation of crystalline phases. Crystalline phases can be identified by comparing to indexed diffraction patterns. The use of an internal standard allows a quantification of the identified phases and in addition of the amorphous phase content. Though amorphous structures cannot be described sufficiently due to the missing requirement of long-range order X-ray.

A powerful technique for the description of network-forming structures is the solid-state nuclear magnetic resonance spectroscopy (NMR) [15]. It can be considered as a complementary method to the PXRD since crystalline and amorphous structures can be assigned. The signal’s full width at half maximum (FWHM) is correlated with the phase crystallinity.

The main restriction in the NMR technique is the requirement of NMR active isotopes of the nuclei of interest. Since common nuclei in materials for refractory applications like ^1^H, ^27^Al, ^29^Si, and ^31^P are accessible, the NMR technique is a powerful tool for phase assignment. The position of the NMR signal, namely the chemical shift, is influenced by the electronic environment of the observed nucleus. With this information direct conclusions of the structures can be generated. The signal width in the solid-state NMR spectra is mainly affected by the magnetic field strength, the nucleus spin, the natural isotope abundance, and the gyromagnetic ratio. Several pulse sequences were developed to increase the resolution of the NMR spectra.

In contrast to high resolution liquid NMR spectroscopy, in solid-state NMR the signal widths are significantly broader due to the impact of the anisotropy of the chemical shift, homonuclear and heteronuclear correlation, and quadrupole interactions. The approach of magic angle spinning at high spinning frequencies and the use of special pulse sequences allows the reduction of signal width increasing the resolution.

For a correct phase assignment signal comparison with databases or literature is required. Since the availability of records of inorganic compounds in databases is limited the measurements need to be extended by other spectroscopic methods that generate information of the existence of functional groups, like FT-IR and Raman spectroscopy.

The combination of PXRD and NMR techniques can be complemented by FTIR and Raman spectroscopy and scanning electron microscopy (SEM). FTIR and Raman spectroscopy are great tools for the identification of individual phosphates and in addition provides information about phase transitions due to temperature-depending measurements. The use of SEM is typical for the optical analysis of the microstructure, combined with energy dispersive X-ray spectroscopy (EDX) the element distribution in the matrix can be investigated.

For a precise and systematical design of a material with tailored properties it is necessary to understand the structure–property relationship. Therefore, complementing the before mentioned structure measurements with additional investigations on macroscopic properties (mechanical, chemical and thermal) is reasonable. This includes for example the investigation of the strength development in dependence of temperature, time or composition via dynamic mechanical analysis (DMA) or cold bending strength measurements.

## 5. Correlation

### 5.1. Strength Development of Phosphate-Bonded Al_2_O_3_-MgAl_2_O_4_ High-Temperature Ceramics

**Figure 4:**
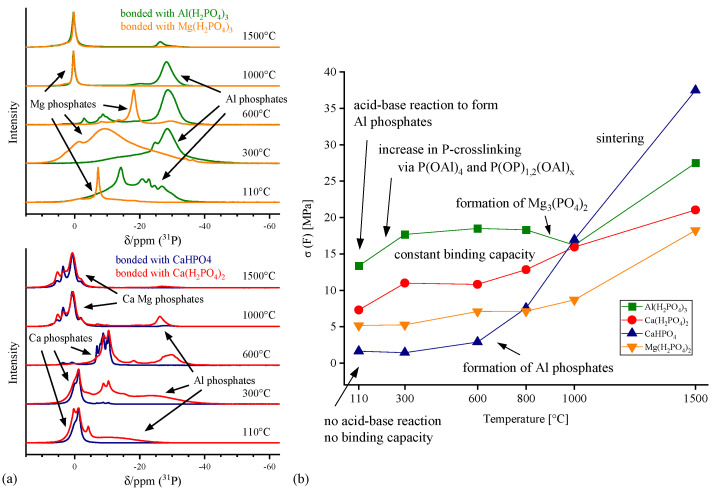
Correlation of the cold modulus of rupture and structural changes in the bonding phase of phosphate bonded Al_2_O_3_MgAl_2_O_4_ ceramics (composition: 85 wt.% Al_2_O_3_, 15 wt.% MgAl_2_O_4_; binder concentration: 3 wt.% P_2_O_5_): (**a**) ^31^P MAS NMR spectra of the phosphate bonded ceramics after thermal treatment (normalized) and (**b**) cold modulus of rupture tests at appropriate temperatures. The strength development of ceramic bodies can be directly correlated with the evolution of an aluminum phosphate bonding network [16].

Generating sufficient early stage strength, that allows ceramic components to be shaped and processed, is of utmost importance in ceramic manufacturing. By adding phosphate binders to the ceramic mix, the bonding strength of ceramics can be improved. Through extensive investigation of structural changes of the bonding phase, differences and changes in the bonding strengths can be explained analytically. For example, structure–property correlations can be achieved by comparing the cold modulus of rupture of phosphate bonded Al_2_O_3_-MgAl_2_O_4_-ceramics with structural changes in the bonding phase after a defined thermal treatment monitored, for instance, by ^31^P MAS NMR (Figure 4).

The strength development of the ceramic components varies depending on the phosphate used. Early stage strength is achieved by applying water soluble phosphates as binders (water solubility at 25 °C: Al(H_2_PO_4_)_3_: >250 g/L; Mg(H_2_PO_4_)_2_: 70 g/L; Ca(H_2_PO_4_)_2_: 20 g/L; CaHPO_4_ < 1 g/L). Further heating generally leads to an increased strength of the components, but the strength developments as well as the end values after thermal treatment at T = 1500 °C vary significantly.

The strength development of the phosphate-bonded ceramics can be explained by evaluating the structure of phosphatic bonding phases. Investigating the phosphate connectivity by solid-state ^31^P NMR allows conclusions to be drawn about the bonding capacity of the developing phosphate structures. It can be stated that the formation of a P-crosslinked aluminum phosphate bonding network leads to an increasing strength of the ceramic components. Aluminum is incorporated in the glass structure and stabilizes crosslinked phosphate bonding networks. Within the refractory body, the binder creates a branched three-dimensional network because of chemical reactions, polymerization and polycondensation. With increasing temperature, the degree of P-crosslinking continuously increases via multiple crosslinking reactions (P–O–Al and P–O–P bonds) within the initialization and network-forming stages The bonding effect is based on adhesive and cohesive forces of the amorphous aluminum phosphate network thus leading to the strength development seen in Figure 4. The development of aluminum phosphates has been verified by solid-state ^27^Al NMR and PXRD investigations.

When using water-insoluble phosphates as binders, no early stage strength is achieved since no acid–base reactions are initiated. However, bonding strength can be generated by heating (T > 600 °C) resulting in a thermally-induced development of aluminum phosphates, thus, bonding strength.

Besides aluminum phosphates, other phosphate species (such as calcium or magnesium phosphates) do not generate bonding effects, since they do not react with ceramic oxides at T < 600 °C and generally are not capable to form broad phosphate networks because of non-bridging-oxygens [10,11,16].

At temperatures T ≥ 600 °C the phosphate phases react with MgAl_2_O_4_ to form Mg_3_(PO_4_)_2_, Ca_x_Mg_y_(PO_4_)_6_ and other crystalline ortho phosphates. The formation of these low-melting high-temperature phases leads to a partial break in the bonding network. Since high temperature processes include the sintering of ceramic particles, in total, temperature treatment at T > 1000 °C leads to a significant increase in bonding strength.

In conclusion, phosphate binders actively influence the chemistry of the ceramic components (e.g., cation exchange, spinel decomposition). By monitoring structural changes in the bonding phase, property developments can be explained. Ultimately, this correlation leads to a better understanding of phosphate bonding mechanisms and, consequently, the mode of action of phosphate binders.

### 5.2. Insights in the Setting Process of Sodium Water Glasses Using a Combination of Dynamic Mechanical Analysis, NMR and PXRD

**Figure 5:**
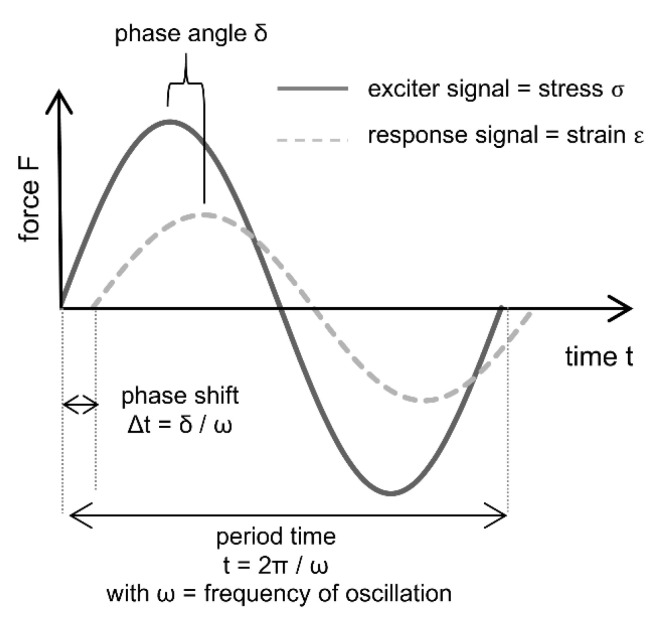
Wave function of exciter and response signal in DMA according to [18].

For the kinetic description of the setting process itself, the dynamic mechanical analysis (DMA) can obtain valuable information by measuring the viscoelastic properties of a material as a function of temperature, frequency and time. This allows time-dependent tracking of the strength development during setting. When combined with the structure information obtained by the above-mentioned methods, insights in the mechanism of the setting process are gained. During the measurement stress is put onto the material by an oscillating movement of a feeler stamp, the so-called exciter signal. This leads to a response signal of the sample, that is material-specific and depends on the viscoelastic behavior (Figure 5). DMA describes the changes in the materials rigidity by the complex modulus of elasticity (MOE), which is composed of the storage modulus E^I^ representing the elastic part of energy and the loss modulus E^II^ standing for the energy that is lost due to plastic deformation. The ratio between E^II^ and E^I^ is defined by the loss factor tanδ [17]. Specifically for the investigations of sol-gel-based binder systems, DMA offers the possibility to determine the gel and the glass point during the setting mechanism [18].

**Figure 6:**
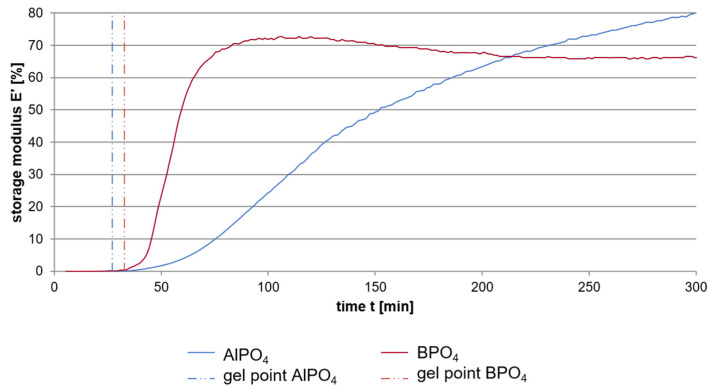
DMA measurements of a sodium water glass hardened with either AlPO_4_ or BPO_4_ [19].

Dynamic Mechanical Analysis investigations of a sol-gel-based pure binder system consisting of a sodium water glass hardened by either aluminum or boron orthophosphate were carried out with the aim to determine the influence of the attendant cation thus leading to a better understanding of the setting process of the alkali silicate systems [19]. The results of the DMA as shown in Figure 6 exhibit the overall setting of the water glass to be much faster when using boron orthophosphate as hardening agent instead of aluminum orthophosphate. Three different phases of the setting process can be discriminated. In an initial phase the storage modulus increases only very moderate until reaching the gel point, which is very similar for both hardeners. This point is followed by a much more intense increase of the storage modulus. This phase now differs highly using the two phosphates. The use of boron phosphate instead of aluminum phosphate leads to a significant faster increase and achievement of the final plateau that can be seen as the third stage of setting.

The increase in the storage modulus in the initial phase is quite similar for both hardeners. Investigations with different hardener portions (10, 20, 30 mass-%) and at different starting temperatures (25 °C, 35 °C, 45 °C) show that this phase of setting is more determined by the temperature and the solid to liquid ratio then by the chemistry of the hardener [20]. Due to the very low solid to liquid ratio in the initial phase, the distances between the engaged species are very high leading to a very low influence of the network building processes of the different phosphate hardeners. Instead, condensation reactions due to the temperature induced release of water and the electrochemical destabilization caused by the change of both the pH value and the surface charge of the silicate particles, determine the setting progress.

**Figure 7:**
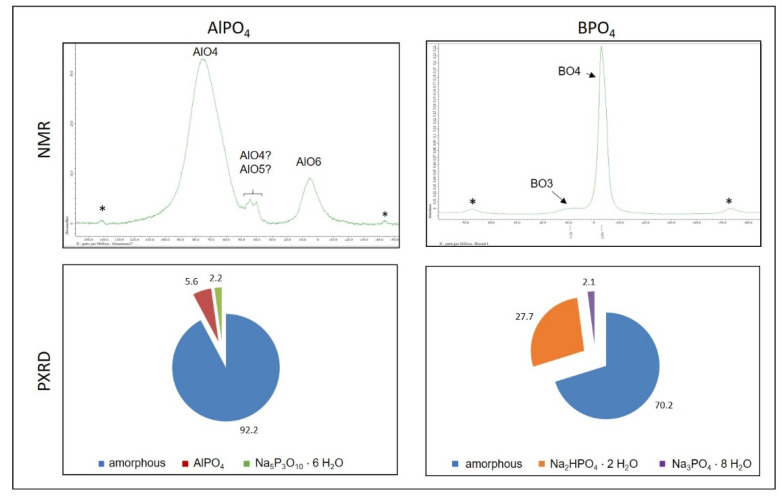
Results of ^27^Al and ^11^B MAS NMR (**1st row**) and PXRD (**2nd row**) measurements of sodium water glass hardened either with AlPO_4_ (**1st column**) or BPO_4_ (**2nd column**) * spinning sidebands [20].

When reaching the gel point, the distances between the different species in the sol are small enough to cause the differences in the hardening mechanisms of aluminum and boron phosphate becoming noticeable: The samples hardened with aluminum phosphate show a significant slower increase in the storage modulus and reach a later but clearly higher final level. NMR of silicon ^29^Si, aluminum ^27^Al and boron ^11^B nuclei explain the reasons for this in the structure of the three-dimensional network, specifically in the coordination of the boron and aluminum compounds integrated in the silicon network. Whereas Al is represented by high coordinated Al species (4,5,6) in the silicon network, boron compounds show a lower grade coordination (3,4) (Figure 7). This is congruent with the observation of higher n-values for Si in the water glass network when using aluminum phosphate instead of boron orthophosphate, indicating a higher linking degree [20]. Hardening with boron orthophosphate leads thus to a fast formed but less interconnected and wide-meshed network. The longer setting of the water glass gained by the use of aluminum orthophosphate results in a higher connected and denser network that leads to the observed higher values of E^I^ and thereby in a higher strength.

PXRD measurements of fully hardened samples (Figure 7, 2nd row) indicate an additional aspect with regard to the different speed of the storage modulus’ increase. Besides the amorphous silicate network both mechanisms lead to the formation of crystalline structures. The use of boron orthophosphate causes the formation of short-chained hydrogen phosphates whereas the addition of the aluminum phosphate lead to the development of water-free long-chained phosphates. In combination with extensive STA measurements, it could be seen, that the two mechanisms cause differences in the release of water. In the case of hardening using AlPO_4_, the samples show a higher weight loss due to the loss of water. However, the results indicated that the hardening mechanism of AlPO_4_ do not generate more free water than the mechanism of BPO_4_, but the mechanism of BPO_4_ to cause a stronger prevention of water what results in the formation of the hydrogen phosphates. The reason for this different retention of water is seen in the inner structure of the originated silicate network with regard to the arrangement of the linking points. The essential aspect here is not the connectivity itself but the location in the network where the linking points are formed. It is known that different energy barriers of the condensation reactions lead to different locations of preferred interaction. This leads to the hypothesis that the network-forming reactions using boron orthophosphate as hardening agent exhibit lower energy barriers and thus induce condensation reactions in the periphery of the originating network. The result is a very fast formation of a far-reaching network that on the one hand hinders free water from release and one the other hand causes a fast hardening and increase in storage modulus. Instead, aluminum phosphate shows a higher energy barrier and thus needs more attempt for a successful condensation. This is even more likely in network areas with a higher density. This results in smaller, but higher linked domains which are surrounded by a certain content of water that thus can easily leave the samples. These structures result in the lower increase in storage modulus of the aluminum phosphate samples.

In an additional test series based on thermally induced linear change, melting behavior indicate a strong impact of the mechanism and the structure on the material properties. Therefore, it is important to identify the determining factors on the setting mechanism for the development of tailor-made material solutions. The outcome of this work points out the combination of the DMA with the methods for structure analysis and the thermo analysis as a powerful tool to connect the setting mechanism itself with the resulting structures and thus with the properties.

### 5.3. Inorganic Phosphate Hardeners in Chemical Setting of Potassium Water Glass

**Figure 8:**
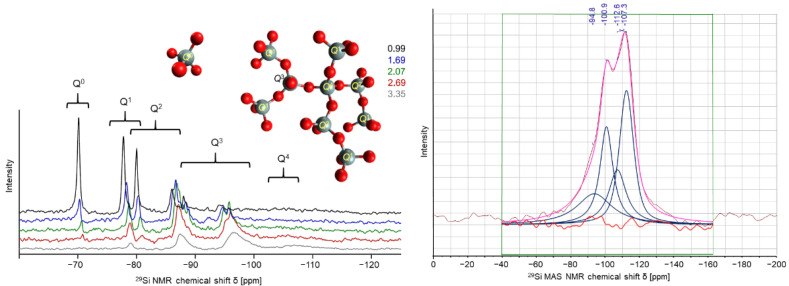
**left**: Q^n^ distribution in liquid ^29^Si NMR with RIDE pulse sequence of pure sodium water glasses, adapted from [21] and **right**: deconvoluted ^29^Si MAS NMR spectra of a potassium water glass with aluminum tetrametaphosphate [22].

In the case of water glass as silicate binders the setting procedure can be conditioned by the admission of inorganic aluminum phosphates as hardening agents. Introducing cyclic aluminum metaphosphate modifications effects significantly the phase formation and the bonding properties, which can be determined by different measurement and spectroscopy techniques like PXRD, solid-state NMR, FT-IR, and Raman spectroscopy. As a monitor of the polycondensation progress of water glass systems the common Q^n^ notation is applied. The Q^n^ distribution in pure sodium water glasses can be considered in Figure 8 left in terms of ^29^Si liquid NMR spectra. The signals for the Q^0^ to Q^4^ can be clearly distinguished and assigned. The spectra in Figure 8 on the right-hand side illustrates the ^29^Si MAS NMR spectra of chemically hardened potassium water glass with aluminum tetrametaphosphate. Due to the nature of solid-state NMR technique and especially of the ^29^Si experiments very broad signals are detected with high overlap. The deconvolution of the signal region resolves four signals, where two can be assigned to Q^3^ and Q^4^ of pure water glass systems. The other two signals are formed by the introduction of aluminum ions into the silicate framework creating alumo-silicate phases. Therefore, it can be demonstrated that the polycondensation shifts the Q^n^ distribution to higher coordinated silicate specimen, since no Q^0^ to Q^2^ signals can be detected.

The impact of the aluminum in the formation of alumo-silicate phases in respect to the alkali modulus (molar ratio of SiO_2_ in respect to M_2_O) of the potassium water glass and the modification of the aluminum metaphosphate on the ^29^Si NMR signals is shown in Table 2. The existence of newly formed Si-O-Al connections is additionally verified spectroscopically by FT-IR and Raman measurements.

Since the mechanical resistance towards deformation is mainly reasoned in the condensation degree of the silicate framework the formation of alumo-silicate sections has an impact on the mechanical properties.

**Figure 9:**
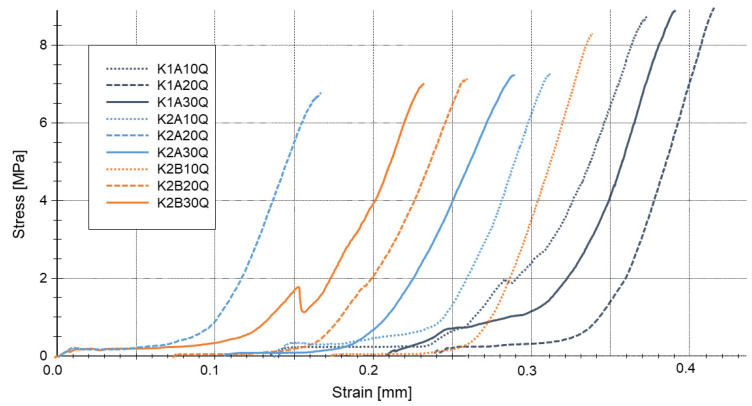
Cold bending strength analysis of potassium water glasses with aluminum metaphosphates (leaned on testing standard DIN EN 853-2-2007). Samples analogous to Table 2 [24].

The quantification of the assigned Q^n^(Al^m^) groups by deconvolution of the solid-state ^29^Si NMR spectra characterizes the degree of alumo-silicate content inside the sample. It can be demonstrated that in case of aluminum hexametaphosphate as hardening agent an increase in alumo-silicate content is detected. Therefore, comparing the alumo-silicate content to the mechanical properties like the cold bending strength and the Young’s modulus by resonance frequency damping analysis (RFDA) a decrease in mechanical stability is obtained (Figure 9). Additionally, the water glass’ alkali modulus influences the mechanical properties, since the samples with a higher K_2_O content show the highest cold bending strength values with the same hardener.

As a result, samples with potassium water glass with a lower alkali modulus and the application of aluminum tetrametaphosphate as hardening agent show the highest values in the cold bending strength tests. This can be correlated to the increase in polycondensation of the silicate framework and a lower degree in formed alumo-silicate phases due to the stronger interception of the aluminum ions by ion-exchange reaction.

Additionally, the solubility of aluminum ions in the alkaline water glass environment and existing ion-exchange reaction of aluminum-ions from the aluminum metaphosphate and the alkali ions of the water glass are mainly responsible for the formation of alumo-silicate phases. Dissolved aluminum ions are the main source of the alumo-silicate formation. The ion exchange reaction reduces the available aluminum ions and therefore reduces its impact on integrating into the silicate framework.

**Figure 10:**
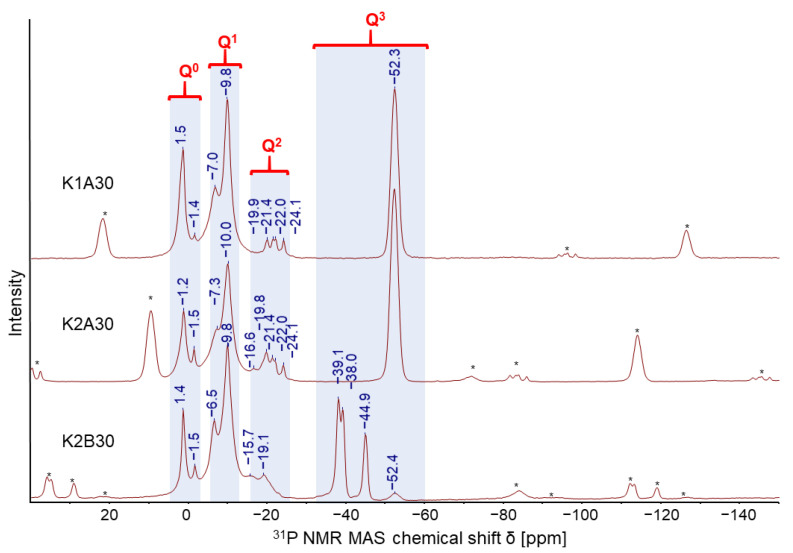
^31^P MAS NMR spectra of two potassium water glasses with different alkali modulus K1 = 3.09, K2 = 3.53 with 30 wt% aluminum tetrametaphosphate KXA30 and aluminum hexametaphosphate K2B30 [22].

For the correct interpretation of the phase formation while setting procedure ^31^P MAS NMR experiments give insights into the depolymerization mechanism of the hardener. In Figure 10 all samples show crystalline potassium hydrogen phosphate as a result of the depolymerizing metaphosphate ring structure. The incrementally decomposition can be found in the of amorphous di- and oligophosphates ranging the Q^1^ area. Crystalline potassium tetrametaphosphate dihydrate was determined as a set of four signals as Q^2^ which proves the existence of an ion-exchange reaction between the aluminum ions from the metaphosphate and the potassium ions from the water glass. As a result, in the case of aluminum tetrametaphosphate as chemical hardener an ion-exchange reaction affects the amount of aluminum ions for a potential formation of alumo-silicate frameworks. Due to the stepwise depolymerization of the cyclic phosphate structure potassium ions are consumed by the formation of alkali phosphate and alkali hydrogen phosphate structures affecting the condensation progress due the lowering of the alkali concentration. As a consequence, the polycondensation of the silicate framework is forced towards highly coordinated networks.

**Figure 11:**
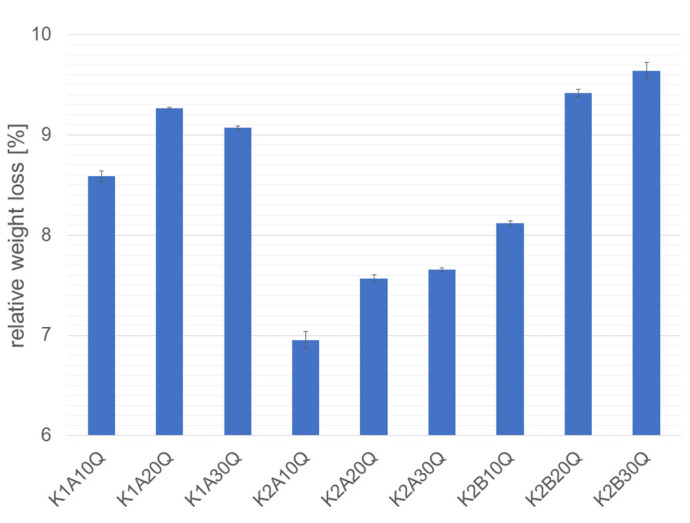
Relative weight loss of potassium water glasses with aluminum metaphosphate hardeners after treatment in hot sulfuric acid according to testing norm DIN 51-102 Part 1. Samples with 30 wt% of potassium water glasses K1 and K2 (alkali modulus K1 < K2), and 10–30 wt% of aluminum metaphosphates A = aluminum tetrametaphosphate, Al_4_(P_4_O_12_)_3_ and B = aluminum hexametaphosphate, Al_2_P_6_O_18_, and 70 wt% quartz as filler [22].

The chemical resistance of water glasses towards acidic attack (see Figure 11) is a function of the alkali concentration of the silicate and alumo-silicate structures, since it is the region of attack. Therefore, the influence of the alkali modulus of the water glass has the strongest impact on the chemical resistance of the water glass system.

Using aluminum metaphosphates as hardening agents for alkali silicate binders increases the chemical stability towards acidic attack, since the available alkali ions are incorporated into alkali phosphate and alkali hydrogen phosphate structures as a result of the incrementally depolymerization of the cyclic metaphosphate structure.

Therefore, aluminum metaphosphate, especially aluminum tetrametaphosphate as hardening agent increases the chemical resistance towards acidic attack in the setting procedure of water glass binder system.

As a summary the chemical setting of potassium water glass binders with aluminum metaphosphates as hardening agents proceeds in a multi-step reaction mechanism involving the incrementally depolymerization of the cyclic metaphosphate structures, the polycondensation of the amorphous three-dimensional silicate network, an ion-exchange reaction, and the incorporation of alkali ions into the silicate framework. All reaction steps mutually influence each other and define the ultimate material properties.

By the structural investigation of the samples in terms of spectroscopic and diffraction techniques, information about the different phase contents are generated while chemical setting. Combining the results of the reaction mechanism with macroscopic properties like cold-bending strength, young’s modulus and chemical resistance towards acidic attack structure–property correlations can be derived. This can be used as a powerful tool in predicting sample properties based on the starting compounds and brings the ability in optimizing the sample matrix for required functionalities.

## 6. Conclusions

Inorganic phosphate binders and phosphate hardened silicates play an important role as refractory binders due to their ability in forming thermally stable phases ranging from room temperature up to T ≤ 1600 °C. Based on the tetrahedral nature of PO_4_ and SiO_4_ units three-dimensional network structures are generated that result in an increased mechanical stability of both the green body as well as the ceramic body after sintering. Analytically, it could be demonstrated that monitoring the multi-step reactions while setting and bonding by the specific combination of complementary structure analysis techniques (PXRD; NMR, Raman Spectroscopy) and macroscopic property investigations (mechanical: DMA cold bending strength analysis), chemical: acid resistance) are the key to the design of tailored material with required properties in respect to the field of application. This correlation allows extracting of the crucial steps for a selective and systematical range of raw material–binder combinations to adjust materials with tailored properties. In contrast to the usual trial and error method this leads to a more efficient approach in material design.

## Figures and Tables

**Table 1:** Bonding systems and their effectivity in respect to the temperature range according to [4].

Bonding System	Effectivity in Respect to the Temperature Range	
	Beginning Approx. °C	End Approx. °C
Ceramic bonding	1000	1200 to 1500
Organic bonding	50	250
Hydraulic bonding	20	500 to 600
Inorganic chemical bonding	20 to 250	1000 to 1450

**Table 2:** Relative intensities of Q^n^ and Q^n^(Al^m^) signals in the ^29^Si MAS NMR spectra of aluminum metaphosphates hardened potassium water glass binders after deconvolution, where Q^n^ stands for the coordination of the silicate group and the m stands for the substituted O-bridged Si-heteroatom connections. Samples with potassium water glasses K1 and K2 (alkali modulus K1 < K2), and 30 wt% of aluminum metaphosphates A = aluminum tetrametaphosphate, Al_4_(P_4_O_12_)_3_ and B = aluminum hexametaphosphate, Al_2_P_6_O_18_. [construct. [22], assignment of the Q^n^(Al)^m^ signals based on studies of [23].

Sample	Relative Intensities of Deconvoluted ^29^Si NMR Signals			
	Q^4^(Al^3^)/Q^3^(Al^1^) at −95 ppm	Q^3^/Q^4^(Al^2^) at −108 ppm	Q^4^(Al^1^) at −108 ppm	Q^4^ at −112 ppm
K1A30	0.23	0.37	0.13	0.28
K2A30	0.23	0.23	0.00	0.55
K2B30	0.20	0.08	0.44	0.29

## Data Availability

The study does not include publicly archived datasets.

## References

[1] Bordia R.K., Kang S.J.L., Olevsky E.A. (2017). Current understanding and future research directions at the onset of the next century of sintering science and technology. J. Am. Ceram. Soc..

[2] Parr C., Auvray J.M., Szepizdyn M., Wöhrmeyer C., Zetterstrom C. (2015). A review of bond systems for monolithic castable refractories. Refract. World Forum.

[3] Girifalco L.A. (2003). Statistical Mechanics of Solids.

[4] Gelsdorf G. (1982). Gliederung, Klassifikation und Prüfung von Feuerbetonen. Keram. Z..

[5] Hewlett P.C. (1998). Lea’s Chemistry of Cement and Concrete.

[6] Viadya S.D., Thakkar N.V. (2001). Study of phase transformations during hydration of rho alumina by combined loss of ignition and X-ray diffraction technique, J. Phys. Chem. Solids.

[7] Kalyoncu R.S. (1982). Chemically Bonded Refractories—A Review of the State of the Art.

[8] Morris J.H., Perkins P.G., Rose A.E.A., Smith W.E. (1977). The chemistry and binding properties of aluminium phosphates. Chem. Soc. Rev..

[9] Kingery W.D. (1950). Fundamental study of phosphate bonding in refractories. J. Am. Ceram. Soc..

[10] Hahn D., Alavi A.M., Quirmbach P. (2021). Powder XRD and ^31^P and ^27^Al solid state MAS NMR investigations of phase transformations in aluminium phosphate bonded Al_2_O_3_-MgAl_2_O_4_ refractories. Mater. Chem. Phys..

[11] Hahn D., Alavi A.M., Hopp V., Quirmbach P. (2021). Phase development of phosphate bonded Al2O3-MgAl2O4 high-temperature ceramics: XRD and solid-state NMR investigations. J. Am. Ceram. Soc..

[12] Derjaguin B., Landau L. (1993). Theory of the stability of strongly charged lyophobic sols and of the adhesion of strongly charged particles in solutions of electrolytes. Prog. Surf. Sci..

[13] Verwey E.J.W., Overbeek J.T.G., van Nes K. (1948). Theory of the Stability of Lyophobic Colloids: The Interaction of Sol Particles Having an Electric Double Layer.

[14] Iler R.K. (1979). The Chemistry of Silica: Solubility, Polymerization, Colloid and Surface Properties and Biochemistry of Silica.

[15] Brow R.K., Kirkpatrick R.J., Turner G.L. (1990). Local structure of xAl_2_O_3_·(1-x)NaPO_3_ glasses: An NMR and XPS study. J. Am. Ceram. Soc..

[16] Hahn D. (2021). Strukturelle Analyse Phosphatischer Bindephasen in Al_2_O_3_-MgAl_2_O_4_-Hochtemperaturkeramiken. Ph.D. Thesis.

[17] Menard K.P. (2008). Dynamic Mechanical Analysis—A Practical Introduction.

[18] Hopp V., Sax A., Helmus D., Quirmbach P. (2019). Adaptation of the dynamic mechanical analysis to determine the gel point during the setting of liquid alkali silicates. Int. J. Appl. Ceram. Technol..

[19] Hopp V., Alavi A.M., Sax A., Quirmbach P. (2020). Influence of Aluminium and Boron Orthophosphate on the Setting and the Resulting Structure of Alkali Silicate Binders for Refractory Application. Ceramics.

[20] Hopp V. (2019). Einfluss von Aluminium- und Bororthophosphat auf die Chemische Härtung von Natron-Wasserglas-Gebundenen Feuerfesten Rieselmassen. Ph.D. Thesis.

[21] Jansson H., Bernin D., Ramser K. (2015). Silicate species of water glass and insights for alkali-activated green cement. AIP Adv..

[22] Alavi A.M. (2019). Einfluss der Struktur von Aluminium-Metaphosphaten auf die Chemische Härtung von Kalium-Wasserglas-Bindern. Ph.D. Thesis.

[23] Thomas J.M., Klinowsky J., Ramadas S., Anderson M.W., Fyfe C.A., Gobbi G.C. (1983). New Approaches to the Structural Characterization of Zeolites: Magic-Angle Spinning NMR (MASNMR). Intrazeolite Chemistry.

[24] Alavi A.M., Breitzke H., Hemberger Y., Sax A., Buntkowsky G., Quirmbach P. (2021). Insights into the mechanism of the chemically initiated setting of potassium silicate solutions with aluminum metaphosphates. Correlation of the structural and macroscopic parameters on the performance of the binding properties. Constr. Build. Mater..

